# Linking Demographic Processes of Juvenile Corals to Benthic Recovery Trajectories in Two Common Reef Habitats

**DOI:** 10.1371/journal.pone.0128535

**Published:** 2015-05-26

**Authors:** Christopher Doropoulos, Selina Ward, George Roff, Manuel González-Rivero, Peter J. Mumby

**Affiliations:** 1 School of Biological Sciences, The University of Queensland, St Lucia, Queensland, Australia; 2 Australian Research Council Centre of Excellence for Coral Reef Studies, St Lucia, Queensland, Australia; 3 Global Change Institute, The University of Queensland, St Lucia, Queensland, Australia; Instituto Español de Oceanografía, SPAIN

## Abstract

Tropical reefs are dynamic ecosystems that host diverse coral assemblages with different life-history strategies. Here, we quantified how juvenile (<50 mm) coral demographics influenced benthic coral structure in reef flat and reef slope habitats on the southern Great Barrier Reef, Australia. Permanent plots and settlement tiles were monitored every six months for three years in each habitat. These environments exhibited profound differences: the reef slope was characterised by 95% less macroalgal cover, and twice the amount of available settlement substrata and rates of coral settlement than the reef flat. Consequently, post-settlement coral survival in the reef slope was substantially higher than that of the reef flat, and resulted in a rapid increase in coral cover from 7 to 31% in 2.5 years. In contrast, coral cover on the reef flat remained low (~10%), whereas macroalgal cover increased from 23 to 45%. A positive stock-recruitment relationship was found in brooding corals in both habitats; however, brooding corals were not directly responsible for the observed changes in coral cover. Rather, the rapid increase on the reef slope resulted from high abundances of broadcast spawning *Acropora* recruits. Incorporating our results into transition matrix models demonstrated that most corals escape mortality once they exceed 50 mm, but for smaller corals mortality in brooders was double those of spawners (i.e. acroporids and massive corals). For corals on the reef flat, sensitivity analysis demonstrated that growth and mortality of larger juveniles (21–50 mm) highly influenced population dynamics; whereas the recruitment, growth and mortality of smaller corals (<20 mm) had the highest influence on reef slope population dynamics. Our results provide insight into the population dynamics and recovery trajectories in disparate reef habitats, and highlight the importance of acroporid recruitment in driving rapid increases in coral cover following large-scale perturbation in reef slope environments.

## Introduction

Coral settlement and post-settlement success can function as demographic bottlenecks to population growth and recovery trajectories in coral reefs environments [1–3]. Following disturbances, growth of remnant colonies and coral recruitment (incorporating settlement and post-settlement survival) drive reef recovery [4]. Remnant growth is often a fast recovery process [5, 6], but mass recruitment is essential to severely disturbed environments when remnant colonies are scarce [7–9]. In combination, recovery following large scale disturbance can be rapid on Indo-Pacific reefs, approximately in a decade [8, 10, 11]. A recent study from the Indian Ocean shows that following the 1998 coral bleaching event that reduced live cover by 90% in the Seychelles, juvenile coral densities of >6.2 individuals m^-2^ were necessary for system recovery to coral dominated states rather than shifting to macro-algal dominated states [12]. Accordingly, understanding how the demographics of coral recruits and juveniles influence recovery trajectories provides the capacity to predict recovery following disturbance.

Many key issues connecting how recruitment influences population maintenance and recovery remain unresolved, including the relationship among new recruits, juveniles, and adult stock; differences in the relative importance of recruitment versus post-recruitment processes in determining population size and structure; whether differences in post-settlement survival alter benthic dynamics; and how life-history strategies influence recovery trajectories [7, 13, 14]. Previous studies from various biogeographic regions have tested hypotheses about the relationships among coral recruits, juveniles, and adult communities [15–17]. Linking the community composition of coral recruits to juvenile and adult communities can be highly ambiguous, whereas similarities between juvenile and adult assemblages often occur [17]. However, this is not always the case and juvenile assemblages do not necessarily reflect the adult community [16]. Juvenile abundances, growth, and mortality can be similar among the same coral taxa between regions with very different adult community composition, suggesting that early recruitment processes (i.e. settlement and post-settlement mortality) and differential adult mortality may structure adult populations [18].

Such unresolved variability among the links between coral recruits, juveniles, and adult benthic community composition can partly be explained by three major factors involving differences in: (1) coral life-history strategies; (2) microhabitat availability and selective larval settlement; and (3) coral post-settlement growth and survival. Corals have two major reproductive modes with contrasting scales of larval development, pelagic duration, and settlement behaviour (reviewed in [19]). Broadcast spawning corals release gametes annually for external fertilisation [20, 21]. Competency is typically optimal around 14 days [22], and settling larvae tend to have specific microhabitat preferences [23, 24]. In contrast, most brooding corals have internal fertilisation and continuous release of competent planulae [19] that often settle within their maternal habitat [25, 26], although long distance dispersal does also occur [26, 27]. Brooder strategies have the advantage of greater resistance to habitat degradation because of their rapid generation times, ability to self-fertilise, and release of mature larvae [19]. However, costs of this weedy life-history strategy include limited colony size (usually smaller than spawners) and inferior competitive ability [28, 29]. Incorporating coral life-history strategies into demographic models provides the means to develop a greater understanding of coral community dynamics [30–34].

Recent work has defined ratios between the density of coral recruits with juveniles over a 15 year time period for Caribbean and Pacific reefs [3]. Yet, studies investigating the role of how post-settlement success drives spatial and temporal variation in coral community structure are needed, best determined by repeated censusing of the same individuals over time [3, 35]. Here, by repeatedly surveying permanent plots, we quantify changes in benthic community structure in reef flat and reef slope habitats over two and half years. We then focus our investigation on the assemblages of juvenile corals in the permanent plots as well as individuals on settlement tiles (from <1 mm to 50 mm) to quantify rates of coral settlement, post-settlement survival, and growth. Finally, we use these vital rates to parameterise transition matrix models to evaluate the contributions of life history, adult stock, and coral demographics to the overall recovery trajectories of two contrasting reef habitats.

## Materials and Methods

### Ethics statement

This research was conducted in the Great Barrier Reef Marine Park, Australia, in accordance with permits issued by the Great Barrier Reef Marine Park Authority (31597.1). No protected species were sampled.

### Site description

The study was conducted at Heron reef, southern Great Barrier Reef (GBR). Heron reef has a large tidal amplitude ~3.0m at the largest spring tide. This drives water flow that changes direction every 6 hours, where it is highest on the reef crest and slope, and least on the inner and mid reef flat [36]. Accordingly, rates of sedimentation on the reef slope are ~4 times higher than the flat, and the range in temperature is greater on the reef flat (17.5–32.7°C) than slope (17.7–29.0°C) (C Doropoulos, *unpublished data*).

Heron reef is frequently subjected to cyclone driven wave disturbances and winter storms [7], as well as coral bleaching events [37]. We selected two sites within no-take marine sanctuary zones: one on the mid-outer southern reef flat (S 23°26.789’, E 151°54.860) and one on the northern reef slope (S 23°26.083’, E 151°56.011). While we did not explicitly include within habitat replication in this study, the sites chosen are from a very well-studied system that demonstrates homogeneity within habitats and heterogeneity between habitats [7, 31, 38]. Instead, we distributed our sampling units (broad benthic surveys, permanent plots and settlement tiles—described in detail below) over a large area within each habitat rather than nesting sites within habitats.

Coral cover at the reef flat site consistently declined from 20% in 1964 to ~2% in 1992, due to multiple cyclone impacts [7] and macroalgal competition [39]. More recently, thermal stress has also resulted in several mild and severe bleaching events [37, 40]. At the reef slope site, coral cover increased from 20 to 60% from 1972 to 1986, after which it declined to ~25% in 1994 due to multiple cyclone impacts [7]. In 2008, the reef slope site experienced extensive local damage during a winter storm event, prior to which coral cover was approximately 50% (S Ward, *personal observation*).

### General protocol

We quantified benthic community cover, rates of coral recruitment, growth and mortality, coral stock-recruitment relationships, and the population dynamics of coral communities found on the reef flat and reef slope. Recruits were defined as corals ≤10 mm, juveniles as corals ≤50 mm in maximum diameter, and adults as corals >50 mm. While such a classification of coral juveniles is somewhat arbitrary, it is a commonly used size classification throughout the coral literature (e.g. [3, 16, 41–43]).

Initially, in August 2009, we quantified the broad benthic characteristics (community cover and juvenile coral structure) of both habitats. Then, we haphazardly marked permanent plots around at least one juvenile at each site and also attached settlement tiles to the plots, which were monitored to quantify coral recruitment, survival, and growth. Plots and tiles were sampled at approximately six month intervals for 2.5 years, from August 2009 to February 2012, resulting in six sampling times and five sampling periods: three from austral winter to austral summer, and two from austral summer to austral winter. Finally, we used the coral demographic rates to parameterise matrix models to understand the functioning of coral juvenile dynamics in two disparate habitats.

### Benthic community structure

The broad benthic community of the reef flat and reef slope was initially quantified at the beginning of the study. In each habitat, 10 replicate 20 m transects were randomly placed and separated by ~4 m. Within each transect, 10 replicate 1 m^2^ quadrats, each with a 10 x 10 grid (i.e. 100 cells), were placed every second metre (10 quadrats per transect), covering a total area of 100 m^2^ in each site. In every cell of a quadrat, the benthic cover was quantified according to whether it was dominated by sand/rock, crustose coralline algae (CCA), epilithic algal matrix (EAM, an early successional mix of thin turfs and CCA), fleshy macroalgae, or live coral.

To periodically quantify benthic community structure the permanent plots in both habitats were photographed every sampling time. Community cover was quantified by classifying the substrate under 100 random points per plot (625 cm^2^) using CPCe [44]. We classified the benthic community as sand, rock, small holes, EAM, CCA, dense turf, fleshy macroalgae, other (includes soft corals, sponges, and giant clams), and live coral. Permanent plots were demarcated by hammering stainless steel tent pegs (30 cm long) into the benthos at each corner of a 25 x 25 cm quadrat. On the reef flat, there were 46 permanent plots with a total of 105 individuals. The plots were generally located on small micro-atolls commonly found in the reef flat, were spaced in an area covering 1200 m^2^, and the depth ranged from ~0.1 m to ~3.0 m (due to tidal amplitude). On the reef slope, there were 36 permanent plots marked around a total of 210 individual recruits. The plots were located within 1.6–4.6 m depth at the most extreme low tide, and were spaced in an area covering approximately 400 m^2^. Similar to other studies from the Indo-Pacific region (e.g. [8, 10, 17, 45]), the dominant coral groups were massives (*Porites* and Faviidae), branching Pocilloporidae (including *Pocillopora*, *Stylophora*, *Seriatopora* [reef slope only]), branching *Isopora*, and branching *Acropora* (reef slope only).

To assess whether the benthic community in the permanent plots was representative of the benthic community in each habitat, community cover was compared among permanent plots and transects recorded from the reef flat and reef slope in August 2009. Data were plotted using multidimensional scaling (MDS) and a correlation vector based on Spearman ranking (>0.6) was added to visualise the relationship among the benthic categories and ordination axes. The data were analysed using ANOSIM, based on a Bray-Curtis dissimilarity matrix. Habitat was fixed, with four levels that included reef flat plot, reef flat broad, reef slope plot, and reef slope broad. MDS and ANOSIM were conducted using Primer-E v6 software [46], and overall, the permanent plots were a good representation of the reef-wide benthic community within each habitat (detailed in Results).

Changes in benthic community cover of the permanent plots was compared between habitats (fixed, 2 levels) over time (random, 6 levels) using permutational multivariate analysis of variance (PERMANOVA) based on a Bray-Curtis dissimilarity matrix. Significant main effects were investigated using pair-wise comparisons. Following this, data were averaged within each habitat x time combination to visualise similarities amongst habitats over time using principal coordinate analysis (PCO). A correlation vector based on Spearman ranking (>0.6) was overlaid on the PCO to visualise the relationship among the benthic categories and ordination axes. PCO and PERMANOVA were conducted using Primer-E v6 software [46] with the PERMANOVA+ extension [47].

### Juvenile coral community structure and growth

To characterise juvenile coral community structure, we used the same transects previously described for the characterisation of the broad benthic community (10 replicate x 20 m transects per habitat). Within each 1 m^2^ quadrat (10 per transect), the abundance and taxa of every juvenile coral (i.e. <50 mm) was quantified. Juvenile abundances were compared between habitats with transects as replicates using a t-test with heteroscedastic variance structure in Microsoft Excel. Juvenile coral community structure was compared between habitats with transects as replicates using PERMANOVA with 999 permutations based on a Bray-Curtis dissimilarity matrix. Homogeneity of multivariate dispersion was not met prior to or following data transformation, tested using PERMDISP, therefore a conservative α value of 0.01 was used to avoid a type I error [48]. PERMANOVA and PERMDISP analyses were conducted using Primer-E v6 software [46] with the PERMANOVA+ extension [47].

In August 2009, every known recruit in the permanent plots was mapped, identified, and the maximum diameter measured to the nearest mm *in situ*. At subsequent sampling times, known individuals were located and remeasured, or marked as dead. Coral recruit growth was quantified using linear extension (mm per 6 months) according to six different size classes (≤10 mm, 11–20 mm, 21–30 mm, 31–40 mm, 41–50 mm, >50 mm) for the four dominant coral groups. Any transition that included recruitment or mortality was not included in growth calculations.

Two mixed effect ANOVAs were conducted for comparisons of coral growth. Firstly, average colony linear extension (mm per 6 months) was compared among taxa (4 levels, fixed) and habitats (2 levels, fixed) with time (5 levels) as a random factor to account for the temporal autocorrelation structure resulting from resampling the same individual over time. Secondly, average colony linear extension (mm per 6 months) was compared among taxa and size classes (6 levels, fixed), again with time included as a random factor to account for temporal autocorrelation. Both analyses were based on Euclidean distance, used 999 permutations of the raw growth data, and significant effects were investigated with pair-wise comparisons. Model simplification occurred by pooling any term that had a negative estimate of components of variation or a *P* value >0.25. Raw data did not conform to homogeneity and could not be transformed due to negative growth values (i.e. shrinkage), so the α value was set to 0.01. Mixed effects ANOVAs were conducted using Primer-E v6 software [46] with the PERMANOVA+ extension [47].

### Rates of coral settlement, recruitment and survival

Settlement tiles were used to capture coral settlement at a finer scale due to the difficulty of seeing very small recruits *in situ*. In August of each year, approximately three months before the major spawning event, a tile pair was firmly attached with cable ties to a corner peg of most permanent plots. However, some tile pairs were lost and not all were retrieved six months following deployment (see S1 Table for full details). Each tile pair consisted of unglazed terracotta tiles that were 5 x 5 cm (100 cm^2^ for each pair). Tile pairs were removed from the permanent plots at every sampling time, transported to the laboratory, held in a large outdoor holding tank with flow-through seawater, and scored using a dissecting microscope. Each new recruit was mapped and the maximum diameter was measured to the nearest 100 μm. Tile pairs with recruits were returned to their original plot and resampled to quantify post-settlement survival. Coral recruitment into the permanent plots was also quantified. The smallest recruits found in the plots were 3 mm on the reef flat and 1 mm on the reef slope.

The numbers of corals recruiting onto the settlement tiles and permanent plots were analysed using a generalised linear model (GLM) with Poisson distribution. Quasi-Poisson distribution was applied to the settlement tile data to account for overdispersion [49]. The total number of new recruits were analysed between habitats (fixed, 2 levels) amongst times (fixed, 5 levels). Survival of those new recruits on the settlement tiles and permanent plots were analysed using the same GLMs, but with binomial distribution, as recruits were either dead or alive following re-sampling. Again, for survival of recruits on the settlement tiles, the data were over dispersed, so a quasi-binomial distribution was used. Generalised models were conducted using nlme [50] in R (version 3.0.2; R Development Core Team 2013).

### Relationship between adult stock and recruitment

Stock-recruitment relationships were investigated by comparing the density of reproductive adults in the permanent plots with the density of recruits in the permanent plots. Colonies were considered reproductive based on size measurements. Brooders were considered reproductive at diameters greater than 70 mm for *Pocillopora* [51], 50 mm for *Stylophora* [52], 80 mm for *Seriatopora* [51], and 40 mm for *Isopora* [53]. For broadcast spawners, colonies were considered reproductive when the diameter was greater than 80 mm for *Porites* [54], 70 mm for Faviidae [54, 55], and 120 mm for *Acropora* [56].

Recruit density was standardised to area of available settlement substrate in the permanent plot, which included rock, EAM, CCA, and small holes (micro-crevices). Unavailable settlement substrata included sand, dense turf algae, macroalgae, other (sponges, giant clam tissue, soft corals), and live corals. The data were analysed using linear mixed effects models, with recruit density the dependent variable, adult density the predictor, and habitat a random factor. Brooder data conformed to normality and homogeneity prior to transformation, but the broadcast spawner data needed square root transformation to meet model assumptions. Generalised mixed effect models were conducted using lme4 [57] in R (version 3.0.2; R Development Core Team 2013).

### Juvenile demographic modelling

**Fig 1:**
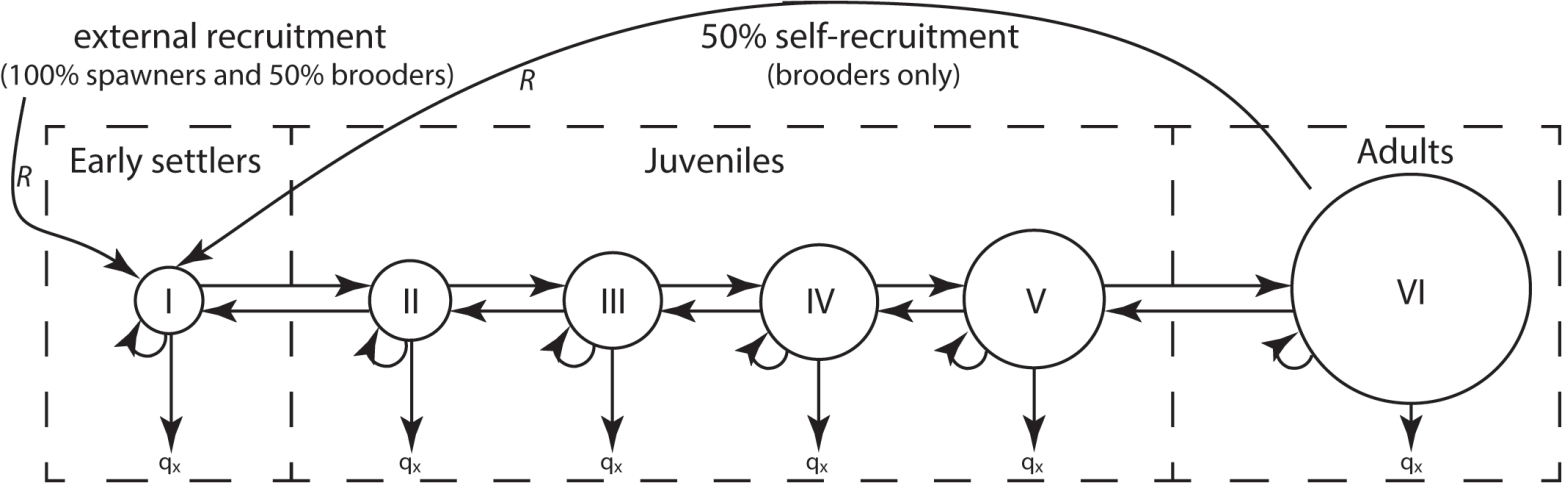
Schematic view of the transition matrices used for brooding and spawning corals. I = 1–10 mm; II = 11–20 mm; III = 21–30 mm; IV = 31–40 mm; V = 41–50 mm; VI = >51 mm; q_x_ = mortality; *R* = recruitment. Brooders are modelled as a semi-closed population, whereas spawners are modelled as a totally open population.

To integrate previous observations of life-history traits and evaluate their importance on the recovery trajectory of corals in the different habitats, we implemented a demographic model that focused on juvenile dynamics (Fig 1). Using transition probabilities for different size-stages of early settler and juvenile corals, the model represents the dynamics of early stage corals and their contribution to early population dynamics. Perturbation analysis and size structure projections were used to evaluate the importance of juvenile size-stages and life-history traits to the recovery of coral populations. Importantly, it is not our intention to use the transition models to predict population dynamics, thus the model focuses on juvenile dynamics and not the entire population. A detailed investigation of all size-stages would be necessary to fully predict overall coral population dynamics. Being centred to high-resolution size-stages of juvenile and early settlers, the model defines the most important drivers of coral population dynamics such as recruitment, early growth and survival.

Transition matrices were separated into five groups based on life-history traits and habitat. Differences in juvenile growth rates were found among brooders and massives on the reef flat, and brooders, massives and fast growing acroporids on the reef slope (see “Juvenile coral community structure and growth” in Results). The reproductive strategy of each coral was retrieved from the literature (see first paragraph of previous section). Moreover, pooling coral taxa based on life-history traits is consistent with other empirical (e.g. [18, 43]), modelling (e.g. [31, 34, 42]), review [58], and meta-analysis [28] studies that find similarities in growth, survival and reproduction within groups, and large differences between groups. Brooder groups combined *Isopora* and Pocilloporidae for the (1) reef flat and (2) reef slope. The spawner groups combined massive *Porites* and Faviidae common to the (3) reef flat and (4) reef slope, and a separate group for (5) spawning *Acropora* unique to the reef slope.

Transition matrices for the juvenile corals at each time period were quantified using the data from the permanent plots, and the mean proportion of the five periods calculated. Individuals were split into six size categories, divided into five equally sized classes for juvenile corals smaller than ≤50 mm, and one class for individuals >50 mm. Size classes include: I = ≤10 mm; II = 11–20 mm; III = 21–30 mm; IV = 31–40 mm; V = 41–50 mm; VI = >50 mm. Transition matrices were fully parameterised using data from the permanent plots and created as follows. Diagonals in the matrices represent the probability of stasis (remaining in the same size class); cells below the diagonal represent the probability of positive growth; and cells above the diagonal represent the probability of negative growth (shrinkage). The bottom row of the matrices represent the probability of mortality (q_x_), and a recruitment vector (*R*) incorporates the number of new recruits entering the populations (e.g. [31, 59]).

To investigate the relative importance of life history traits on the population dynamics of juvenile corals, we centred a perturbation analysis on earlier transitions [60], rather than λ for stable populations [61]. Sensitivity analyses used a standardised distance metric that compared the abundance of individuals in each size class following the projections of an unperturbed population versus a perturbed population. Here, each cell in the transition matrix was perturbed by a 10% increment of the observed value and the residual sum of squares (RSS) from each cell in the unperturbed population was calculated. RSS refers to the standardised difference between projected numbers of individuals at each size class on each perturbed scenario against the projected size structure using the observed parameters (unperturbed). This was converted into a relative scale between 0 to 1, with 0 having the least effect and 1 having the most effect. Projections were based on the transition matrices after 20 time steps (i.e. 10 years).

Different sensitivity analyses were used for brooder and broadcast spawner reproductive modes, because a stock recruitment relationship was discovered for brooders in both habitats, but not for spawners (see *Stock and recruitment* in the *Results* section). For brooders, matrices were modelled as a semi-closed population based on our stock-recruitment data, meaning that 50% of recruits in the first four size classes came from local sexually mature size class (>50 mm), and partial larval supply came from outside our sample population. The exact value of 50% is somewhat arbitrary because of the varying values found in the literature about brooder populations (e.g. [27, 62]). However, it is a conservative approach that recognises a percentage of self-recruitment given by stock fecundity. In contrast, broadcast spawners were modelled as totally open populations (e.g. [59]). Matrix modelling and analysis was conducted using MATLAB (version 7.14.0.739; MathWorks 2012).

## Results

### Trends in benthic community structure

At the beginning of the study (August 2009), the broad benthic community on the reef flat was characterised by sand (~50%), fleshy macroalgae (~20%), and low coral cover (10%). The reef slope was characterised by high EAM (~70%) and low coral cover (18%). The permanent plots represented the reef-wide benthic composition faithfully (S1 Fig), with no significant differences between the community structure in the permanent plots and broad scale surveys within each habitat (S2 Table).

**Fig 2:**
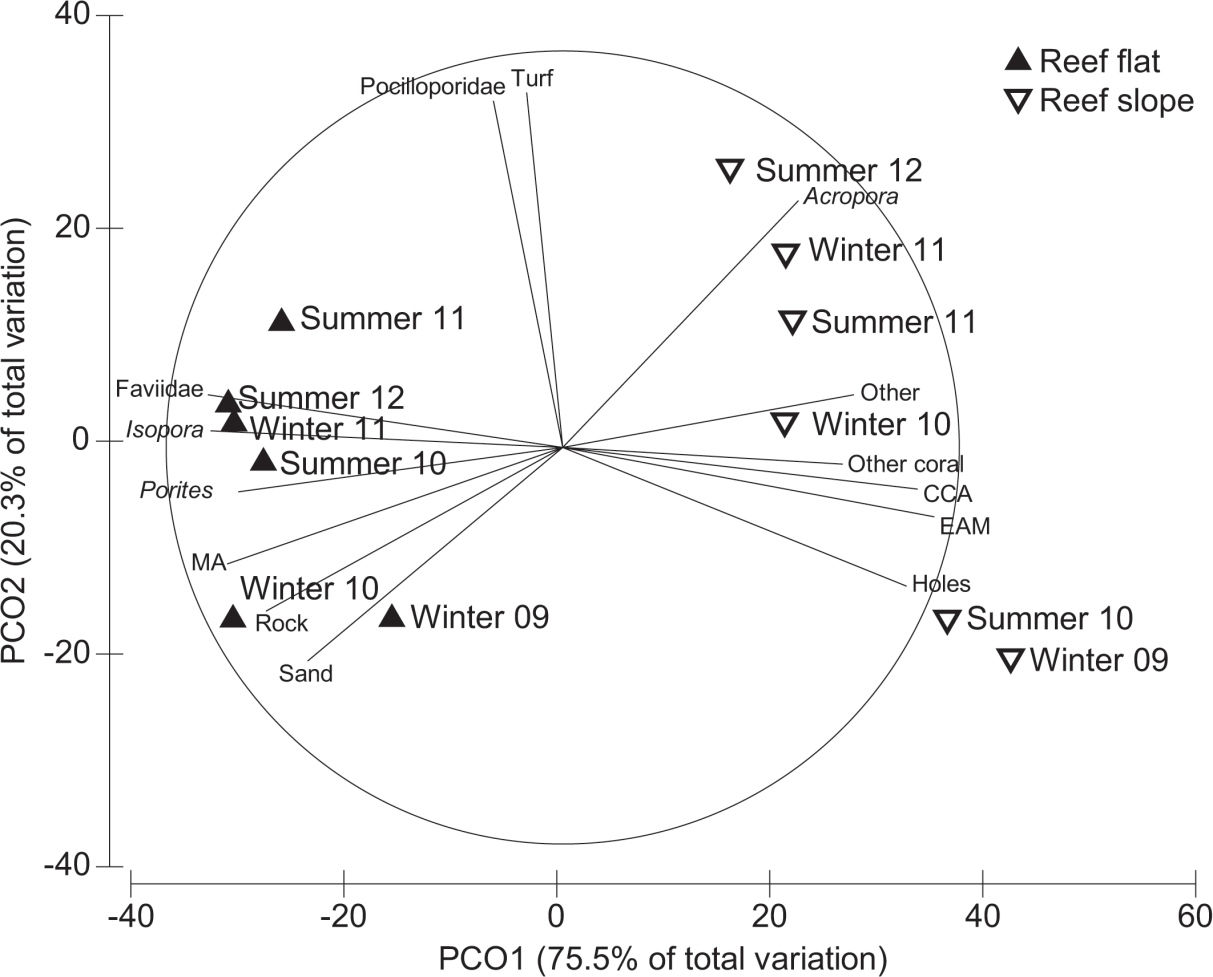
Principal coordinates analysis (PCO) of benthic community cover at reef flat and reef slope habitats from August 2009 to February 2012. Vector overlay represents correlations >0.6 based on Spearman ranking. Upward facing solid triangles = reef flat; downward facing hollow triangles = reef slope. EAM = epilithic algal matrix; CCA = crustose coralline algae; Turf = dense turf algae; MA = fleshy macroalgae; Other = soft coral, sponge, *Tridacna*, unknown.

**Fig 3:**
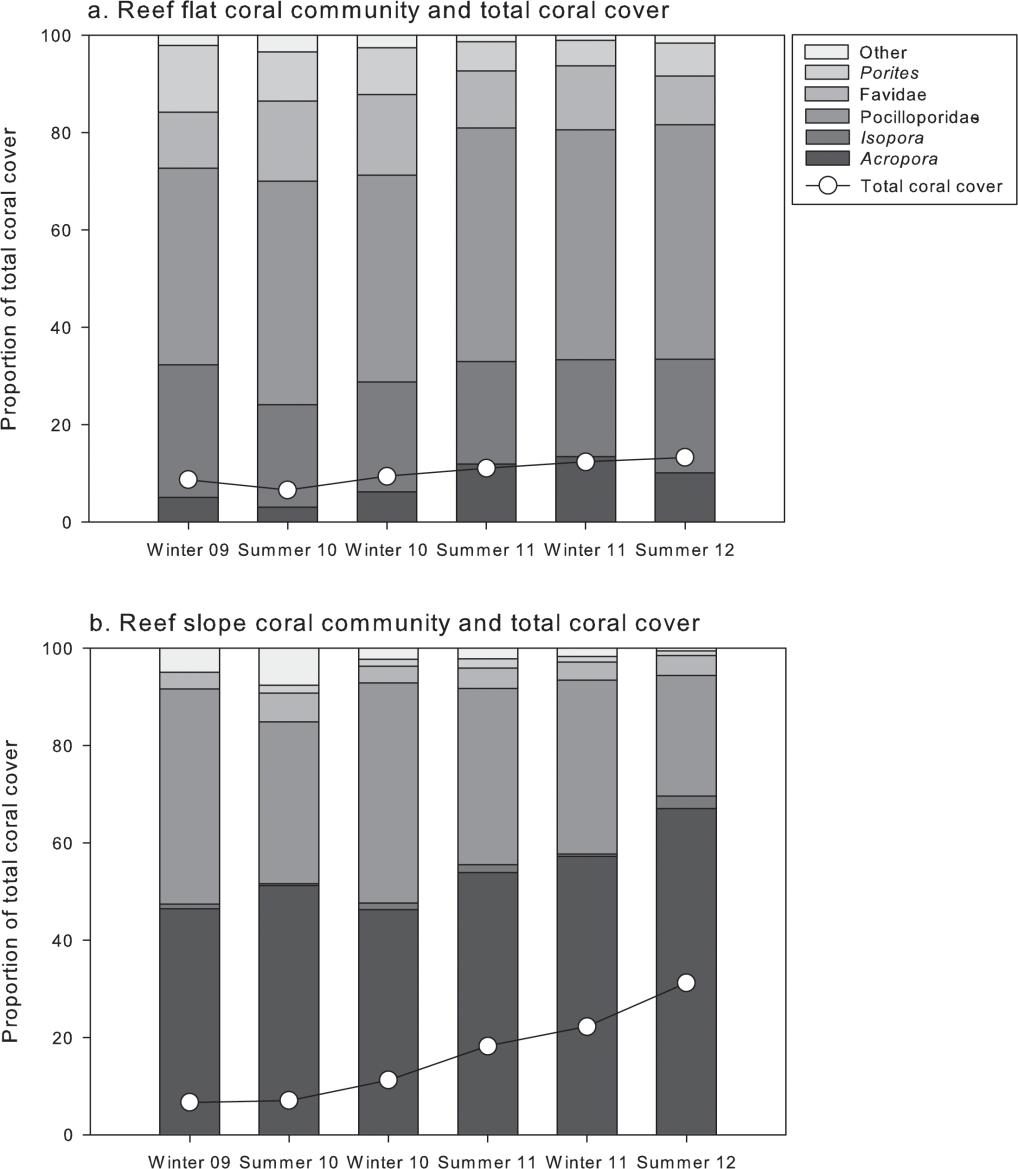
Total (line) and proportional (stacked bars) coral cover at (a) reef flat and (b) reef slope habitats from August 2009 to February 2012.

Community structure varied over time in the permanent plots, but remained distinct between habitats (Fig 2; S3 Table). Both habitats had decreasing EAM and CCA cover, and increasing algal turf cover (S2 Fig). Macroalgal cover increased from 23 to 45% on the reef flat but remained at ~3% on the reef slope (S2 Fig). Coral cover increased 3-fold in 2.5 years on the reef slope from 7% to 31%, whereas it only exhibited modest changes on the reef flat, increasing from 9% to 13% (Fig 3; S2 Fig).

The increase in coral cover on the reef slope was largely driven by *Acropora*, which increased in proportional cover from 46 to 67% of the coral assemblage (Fig 3B). In contrast, *Acropora* was largely absent from permanent plots on the reef flat. Pocilloporidae dominated the reef flat coral assemblage and its proportional cover increased from 40 to 48%, whereas it decreased from 44 to 25% proportional cover in the reef slope. The reef flat coral assemblage also comprised 23% *Isopora*, and 17% massive *Porites* and Faviidae (Fig 3A).

### Juvenile coral community structure and growth

**Fig 4:**
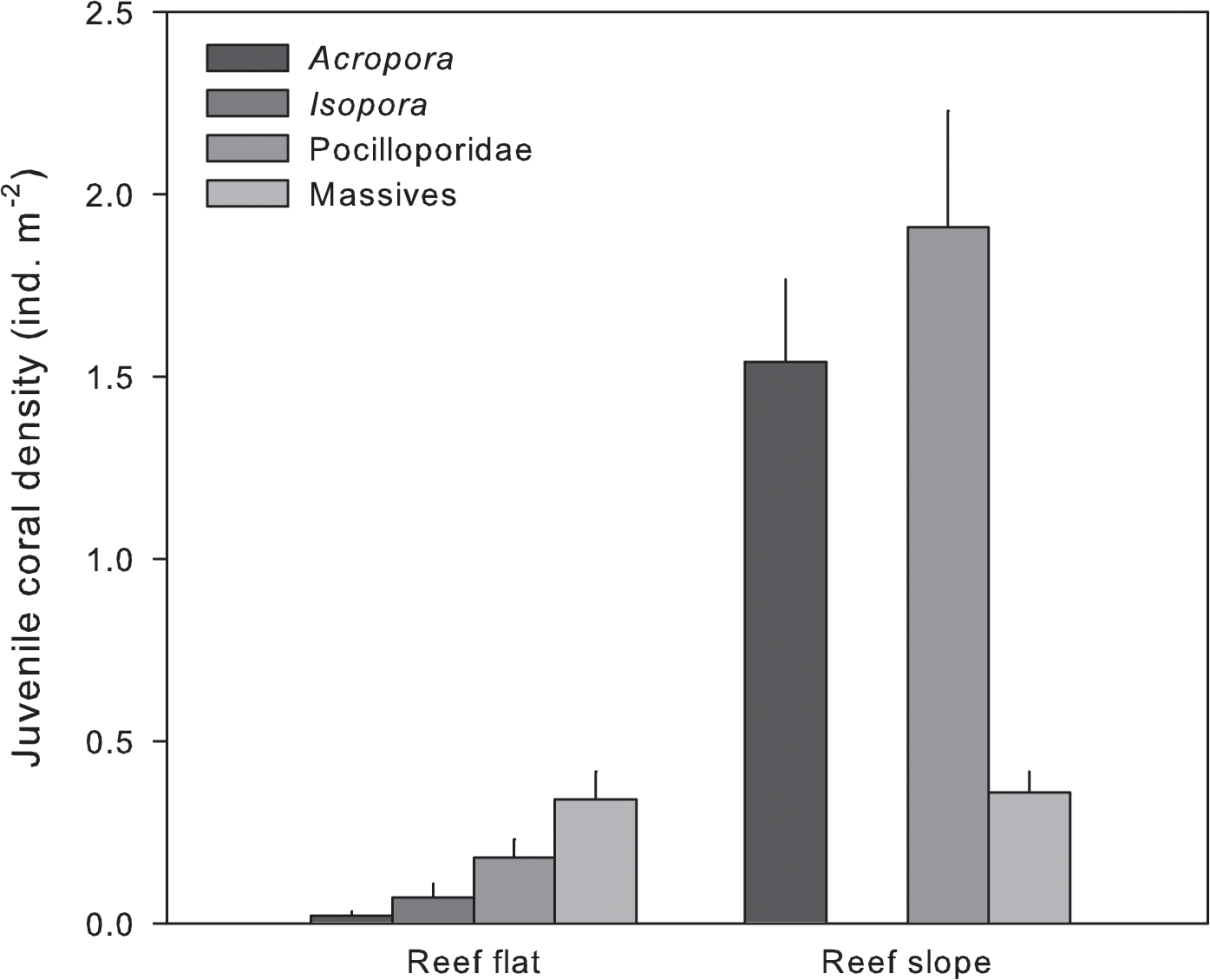
Juvenile coral community structure (individuals m^-2^ + SEM) at reef flat and reef slope habitats in August 2009. Massive = *Porites* and Faviidae.

Juvenile coral abundance was 6 times higher on the reef slope compared to the reef flat (*P* < 0.001), with mean abundances 0.6 individuals m^-2^ (±0.1 SEM) and 3.8 individuals m^-2^ (±0.4 SEM), respectively (Fig 4). Juvenile coral community structure also significantly differed between habitats (*P* < 0.001), due to the dominance of massive corals (56%) on the reef flat compared to *Acropora* (40%) on the reef slope. The proportional abundance of Pocilloporidae juveniles was high in both habitats (30% on reef flat, 50% on reef slope).

**Fig 5:**
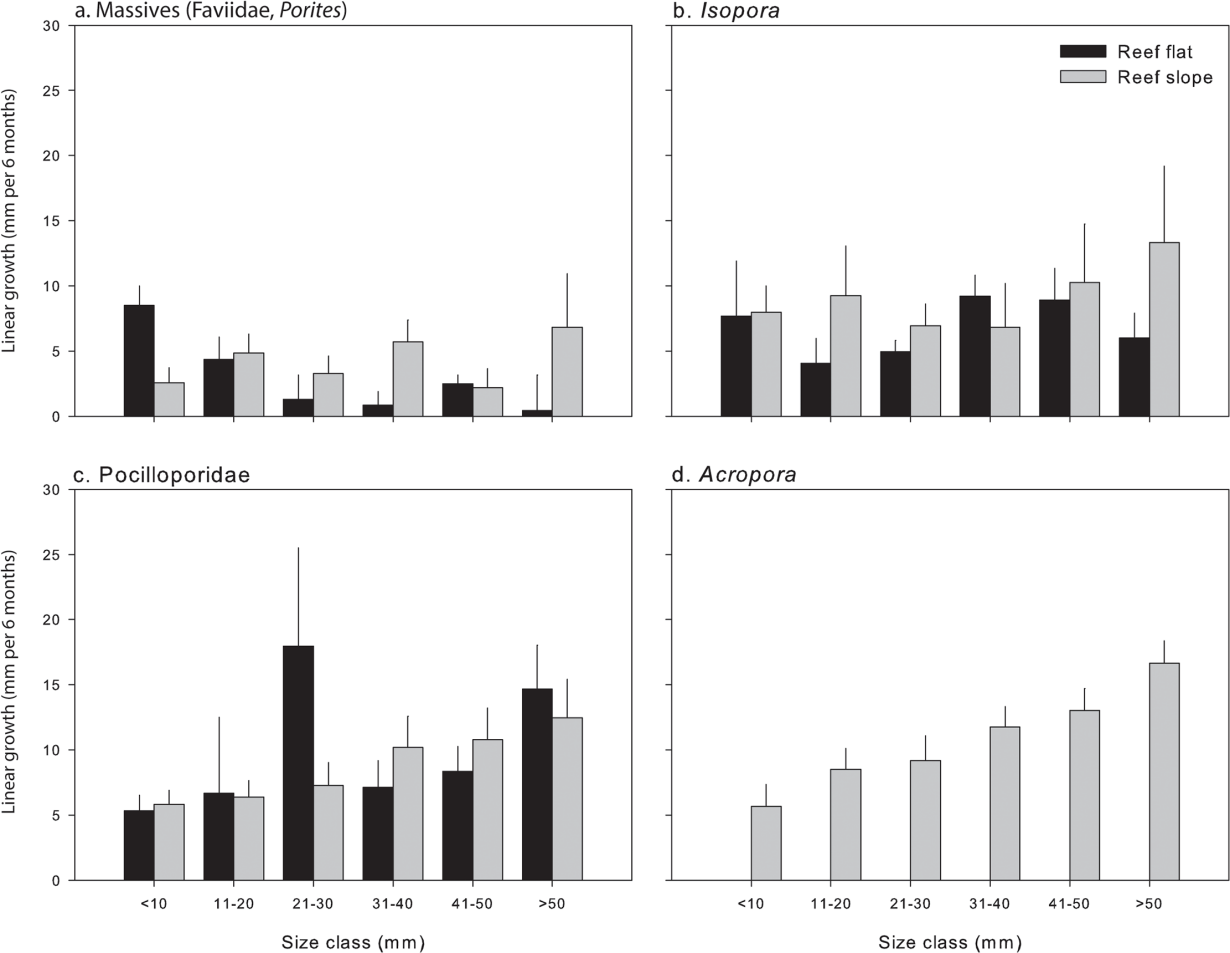
Linear growth (mm per 6 months + SEM) of juvenile (a) Massive (Faviidae, *Porites*), (b) *Isopora*, (c) Pocilloporidae, and (d) *Acropora* corals at reef flat and reef slope habitats based on six size classes. The maximum diameter of corals was measured every six months from August 2009 to February 2012.

Juvenile coral growth rates differed among different taxa within each habitat (taxa x habitat: *P* < 0.01; Fig 5; S4 Table). On the reef flat, linear growth rates significantly differed between all taxa, with massive corals having the lowest growth (1.5 mm per 6 months), *Isopora* having a medium growth rate (6.5 mm per 6 months), and Pocilloporidae having the fastest growth (11.2 mm per 6 months). On the reef slope, massive corals also had the slowest growth (3.9 mm per 6 months), *Isopora* and Pocilloporidae had similar growth rates (8.7 mm per 6 months), and *Acropora* had the highest growth rates (12.2 mm per 6 months). There were no significant differences in growth rates for each coral group between habitats.

Growth rates varied among size class x taxa (*P* < 0.01; S5 Table). Within size classes, there were no significant differences in growth rates among taxa for the smallest recruits <20 mm, apart from *Acropora* that grew 2-times faster than massive corals for individuals 11–20 mm. However, differences in growth between taxa emerged for corals >21 mm at which massive corals grew consistently slower than all other coral taxa. The growth of *Isopora*, Pocilloporidae, and *Acropora* only significantly differed for individuals >50 mm at which *Acropora* grew 1.4-times faster than Pocilloporidae that grew 2-times faster than *Isopora*. Within coral taxa, massive corals (Fig 5A) and *Isopora* (Fig 5B) generally had similar growth rates among size classes. In contrast, growth rates of Pocilloporidae (Fig 5C) and *Acropora* (Fig 5D) generally increased with increasing size.

### Rates of coral settlement, recruitment and survival

**Fig 6:**
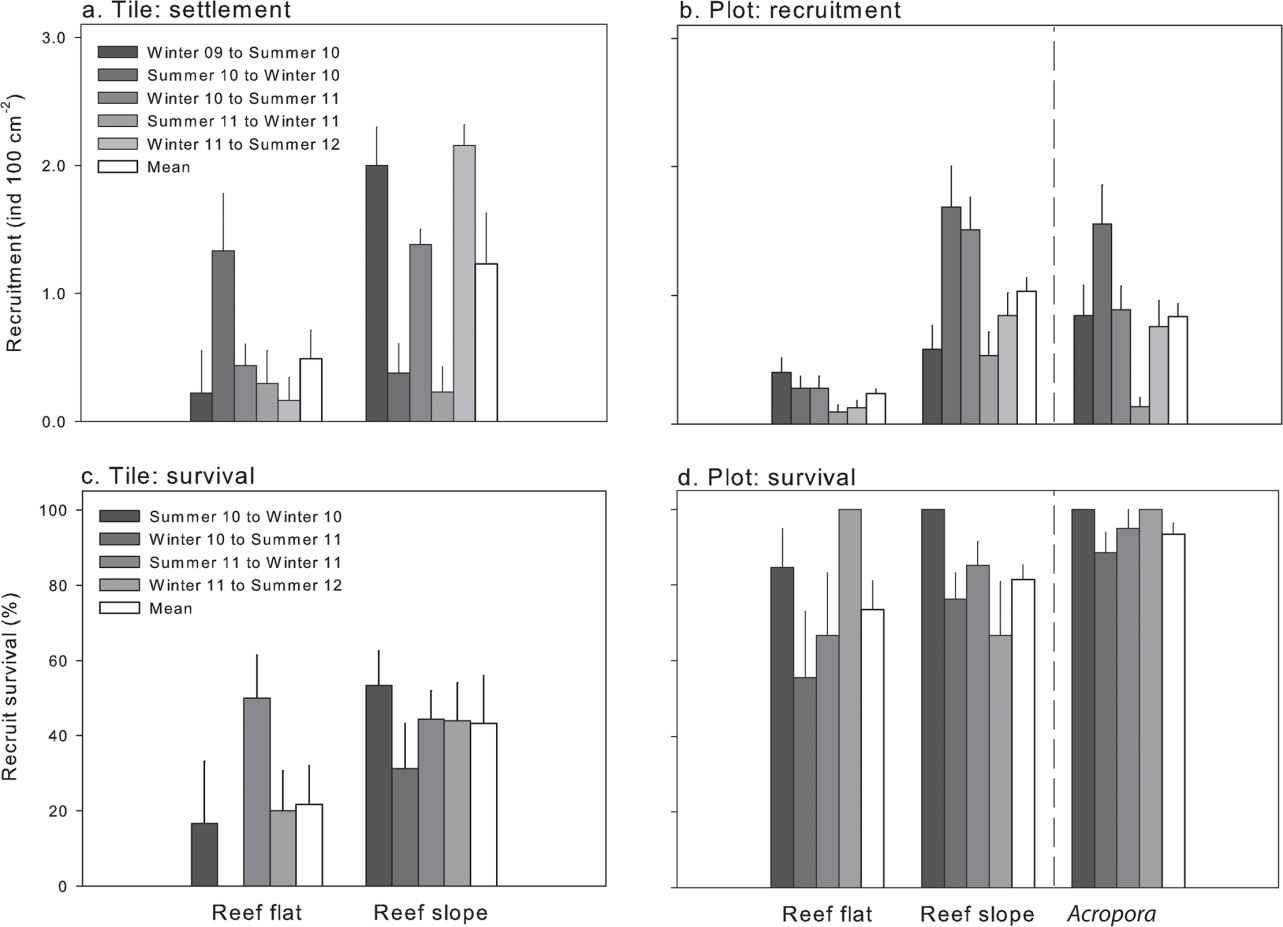
Coral recruitment (a, b) and recruit survivorship (c, d) onto settlement tiles (a, c) and permanent plots (b, d) in reef flat and reef slope habitats every six months from August 2009 to February 2012. Recruitment was quantified as the mean number (+ SEM) of new recruits 100 cm^**-2**^ per 6 months. Survivorship is the mean % (+ SEM) of those recruits that survived. Winter = August; summer = February (except summer 2011 = April). Note the different scales on the y axes.

The mean size of corals settled to tiles was 2.6 (± 2.8 SD) mm. Coral settlement to tiles on the reef slope was double that of the reef flat. Mean settlement was 1.2 and 0.5 individuals 100 cm^-2^ per 6 months on the reef slope and flat, respectively (Fig 6A). However, significant inter-habitat differences in settlement only occurred after major broadcast spawning events, where it was consistently higher in the reef slope (winter to summer; habitat x time, *P* = 0.002). The average survival of recruits on settlement tiles was also 2-fold higher on the reef slope (43%) compared to the reef flat (22%; Fig 6C), yet no significant differences in survival were detected between habitats (*P* = 0.10) and time clearly did not influence post-settlement survival (*P* = 0.22).

The mean size of corals recruiting to the reef itself (i.e. to natural substratum) was 15.1 (± 8.6 SD) mm. Coral recruitment was 4-fold higher on the reef slope compared to the reef flat (Fig 6B). An average of 0.2 individuals 100 cm^-2^ per 6 months recruited to the reef flat, whereas 1.0 and 0.8 individuals 100 cm^-2^ month^-6^ recruited to the reef slope for taxa common to both habitats and *Acropora* unique to the reef slope, respectively. A significant habitat x time interaction was detected (*P* = 0.04), but with no consistent patterns related to time (e.g. post-spawning). The survival of new recruits in the permanent plots (Fig 6D) was more than double that of those settling on the tiles (Fig 6C), and there were significant main effects of habitat (*P* = 0.004) and time (*P* = 0.02) on recruit survival in the permanent plots. Mean survival was significantly higher for new *Acropora* recruits unique to the reef slope (94%) compared to taxa common to both habitats on the reef slope (81%) and reef flat (74%). There were no consistent patterns related to time, apart from significantly lower survival from winter 10-summer 11 compared to summer 11-winter 11.

### Relationship between adult coral stock and recruitment

**Fig 7:**
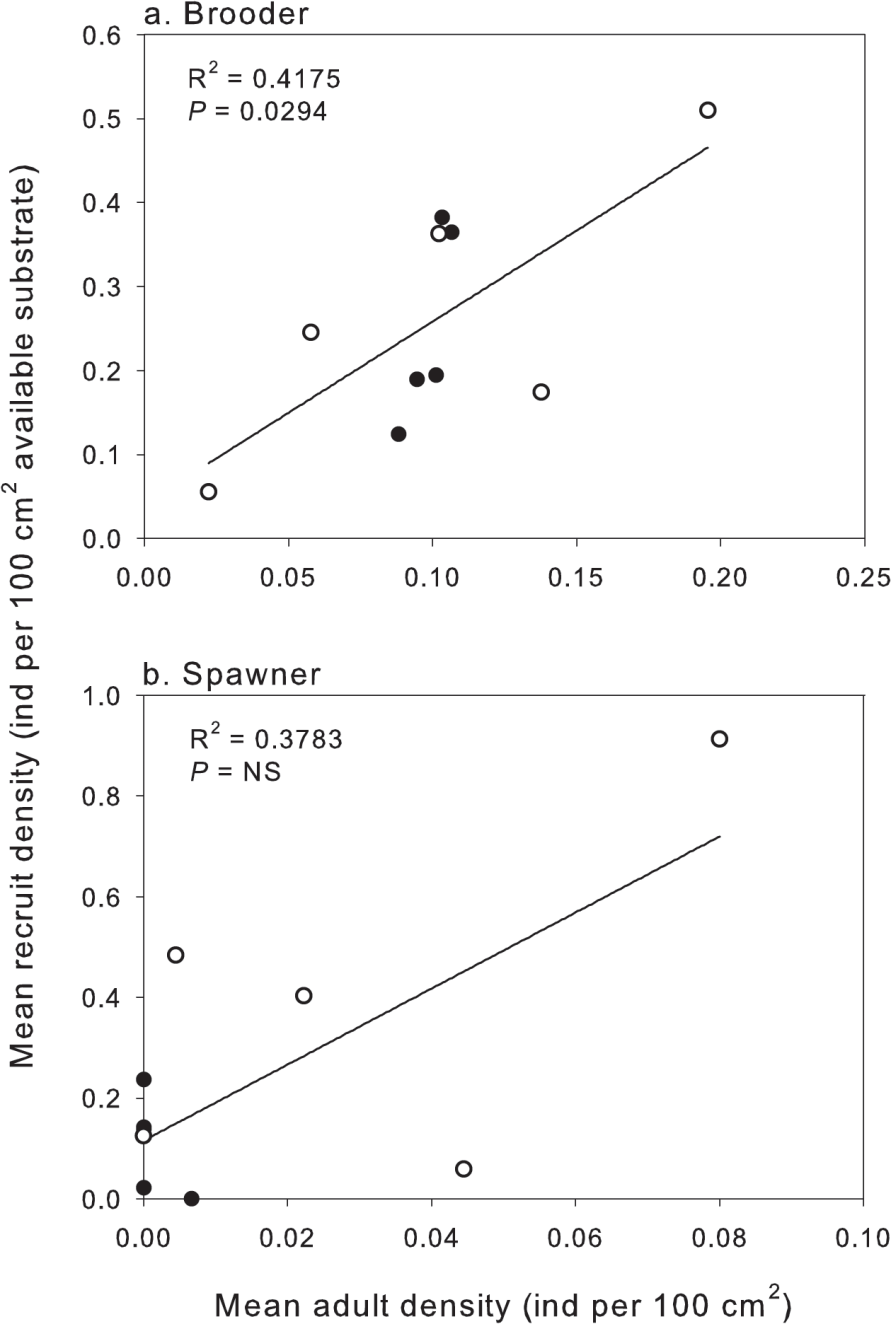
Stock-recruitment relationship between the abundance of individual mature coral colonies and coral recruits in permanent plots depending on whether the colonies (a) brood larvae or (b) spawn unfertilised gametes. Closed circles represent samples from the reef flat and open circles from the reef slope. Recruit density was standardised to the area of available settlement substrata in the permanent plot (see *Methods* for available and unavailable settlement substrate classifications). Note the different scales on x and y axes.

A positive stock-recruitment relationship was found in both habitats for corals that brood their larvae (Fig 7A). This stock-recruitment function occurred over time, reflecting an increased rate of recruitment as the adult stock of brooders increased (positive increase in the abundance of brooder recruits with adult stock, R^2^ = 0.42; *P* = 0.03). However, there was no evidence of a stock-recruitment relationship for broadcast spawners (Fig 7B; relationship between recruitment and stock R^2^ = 0.38; *P* = 0.76).

### Juvenile demographic modelling

Most corals (>93%) escaped mortality once they exceeded 50 mm irrespective of life-history and habitat. Nevertheless, when considering the mortality of juveniles (<50 mm), distinct differences were apparent. Mortality in the smaller size classes (<20 mm) of brooders (*Isopora*, Pocilloporidae; Table 1) were double that of spawning *Acropora* (Table 2C) and massive corals (*Porites*, Faviidae; Table 2A and 2B). Furthermore, juvenile mortality was 2.5-times higher on the reef flat (Table 1A and Table 2A) than the reef slope (Table 1B and Table 2B and 2C), and was intensified for larger size classes on the reef flat and smaller size classes on the reef slope. Coral shrinkage was minor and rarely exceeded 10% in any size class or life-history group.

**Table 1:** Transition matrices for brooder corals on the (a) reef flat (*Isopora*, *Pocillopora*, *Stylophora*) and (b) reef slope (*Isopora*, *Pocillopora*, *Stylophora*, *Seriatopora*).

	a. Reef flat (brooders)						b. Reef slope (brooders)					
Size class	I	II	III	IV	V	VI	I	II	III	IV	V	VI
I	0.28	0.03	0.00	0.00	0.00	0.00	0.27	0.02	0.01	0.00	0.00	0.00
II	0.28	0.31	0.02	0.00	0.00	0.01	0.30	0.35	0.08	0.03	0.00	0.02
III	0.00	0.20	0.27	0.00	0.02	0.00	0.06	0.26	0.31	0.07	0.04	0.01
IV	0.00	0.02	0.32	0.35	0.03	0.01	0.01	0.11	0.32	0.18	0.08	0.01
V	0.00	0.02	0.06	0.23	0.17	0.04	0.00	0.02	0.16	0.43	0.22	0.03
VI	0.00	0.00	0.03	0.08	0.48	0.87	0.00	0.00	0.03	0.27	0.60	0.93
*q*_*x*_	0.43	0.42	0.30	0.34	0.30	0.07	0.37	0.23	0.09	0.03	0.05	0.02
*R*	12	8	4	2	0	0	45	21	8	5	0	0
*n*	19	28	32	41	43	134	96	132	102	68	58	121

Each cell in the matrix is the mean proportion of a transition every six months for five time periods. Size classes are: I = 1–10 mm; II = 11–20 mm; III = 21–30 mm; IV = 31–40 mm; V = 41–50 mm; VI = >51 mm. *q*_*x*_ = mean mortality. *n* = total number of individuals (excluding new recruits) in that size class for the entire study; *R* = total recruitment in that size class for the entire study.

**Table 2:** Transition matrices for the broadcast spawning corals of shared massives (*Porites*, Faviidae) on the (a) reef flat and (b) reef slope, and (c) *Acropora* taxa (*Acropora* spp, *A*. *humilis*, *A*. *hyacinthus*, *A*. *nasuta*) on the reef slope.

	a. Reef flat (spawners, massives)						b. Reef slope (spawners, massives)						c. Reef slope (spawners, *Acropora*)					
Size class	I	II	III	IV	V	VI	I	II	III	IV	V	VI	I	II	III	IV	V	VI
I	0.45	0.00	0.03	0.00	0.00	0.00	0.49	0.03	0.02	0.00	0.00	0.00	0.40	0.03	0.01	0.00	0.00	0.00
II	0.25	0.57	0.07	0.00	0.00	0.02	0.40	0.47	0.10	0.00	0.03	0.00	0.36	0.29	0.07	0.02	0.00	0.00
III	0.05	0.19	0.56	0.16	0.03	0.02	0.00	0.37	0.54	0.06	0.00	0.00	0.13	0.32	0.27	0.02	0.01	0.00
IV	0.00	0.04	0.19	0.56	0.00	0.00	0.00	0.06	0.26	0.58	0.00	0.00	0.02	0.18	0.38	0.17	0.02	0.00
V	0.00	0.00	0.03	0.11	0.57	0.11	0.00	0.00	0.00	0.19	0.70	0.07	0.00	0.02	0.17	0.35	0.17	0.00
VI	0.00	0.03	0.02	0.08	0.27	0.85	0.00	0.00	0.02	0.14	0.27	0.93	0.00	0.00	0.05	0.33	0.71	0.97
*q*_*x*_	0.25	0.17	0.12	0.10	0.13	0.00	0.11	0.07	0.05	0.03	0.00	0.00	0.10	0.15	0.05	0.11	0.09	0.02
*R*	1	6	3	2	0	0	12	15	**7**	4	0	0	23	49	18	3	0	1
*n*	8	28	44	41	22	45	20	51	48	21	10	8	44	120	95	78	61	154

Each cell in the matrix is the mean proportion of a transition every six months for five time periods. See Table 1 for size class and label definitions.

**Fig 8:**
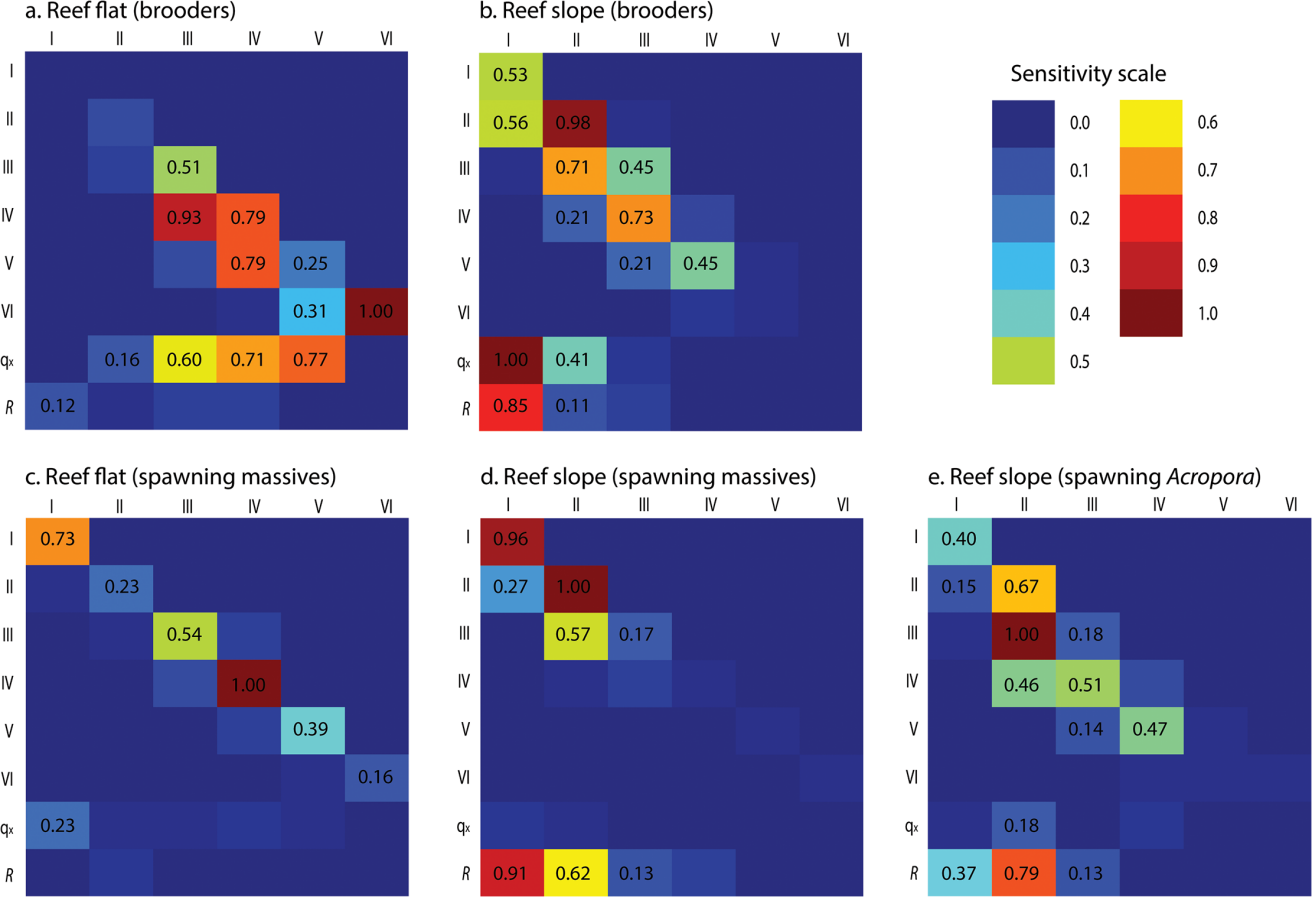
Sensitivity analyses of transition matrices for brooder corals on the (a) reef flat and (b) reef slope, for spawning massives on the (c) reef flat and (d) reef slope, and spawning (e) *Acropora* on the reef slope. The values displayed are a relative measure of the magnitude of the deviance that the perturbed assemblages (10%) have from the unperturbed assemblages at every transition. Text within a cell is only displayed for sensitivity values >0.1.

Sensitivity analyses demonstrated that the population of brooding corals on the reef flat was most sensitive to perturbation affecting stasis (diagonal vector), growth and mortality of the larger size classes from 20–50 mm (Fig 8A). In contrast, the population of brooders on the reef slope were highly sensitive to perturbation affecting recruitment, mortality, and stasis and growth of the smallest size class <20 mm (Fig 8B).

Massive corals in both habitats had low recruitment, slow growth, and low mortality among all size classes (Table 2A and 2B). Sensitivity analysis demonstrated that the population on the reef flat was most sensitive to perturbation affecting stasis among all size classes (Fig 8C), whereas the assemblage on the reef slope was highly sensitive to perturbation that affected recruitment, survival, and growth of individuals <20 mm (Fig 8D).

The population of *Acropora* on the reef slope generally had low mortality and high positive growth among all size classes (Table 2C). Sensitivity analysis demonstrated recruitment, and stasis and growth of the smallest size classes (<20 mm) were highly sensitive to perturbation (Fig 8E).

## Discussion

Our study demonstrates how juvenile coral demographics can influence recovery trajectories in distinct habitats, driven by marked differences in patterns of settlement, survivorship, and coral identity. Over time, we found a significant stock-recruitment relationship with brooders, but not with broadcast spawners; yet, it was a combination of recruitment, rapid growth and high survival of spawned *Acropora* that drove the increase in coral cover on the reef slope. Recruitment rates on the reef slope were more than double those on the reef flat, and these were driven by spawners, at two scales of resolution; early settlement captured by the settlement tiles, and recruitment of new individuals to the reef benthos. Settlement by spawning corals accounts for the highest proportion of coral recruitment on Pacific reefs [63], which is not surprising given their dominance in the adult community (~80%) [58]. Mortality of recruits on settlement tiles (~70%) was high in both habitats, highlighting the first post-settlement recruitment bottleneck [43, 64–68]. Once recruits reached larger size classes, critical differences in mortality between the habitats and coral taxa became apparent, as has been observed with *Acropora* and *Pocillopora* corals in Moorea [69]. Brooded corals had higher mortality rates than spawners, and corals on the reef slope generally escaped mortality once they reached 20 mm. The size-escape of coral recruits was delayed on the reef flat, having a critical effect on the population until they grew larger than 40 mm, most likely driven by competition with macroalgae (e.g. [70, 71]) and sediment smothering (e.g. [72]), both of which are dominant components of the benthic community in this habitat.

While many studies have modelled coral population and community dynamics on Caribbean (e.g. [2, 59, 73]) and Indo-Pacific (e.g. [31, 34, 42, 55, 74]) reefs to understand succession and shifts in community structure following disturbance, none has focussed on the dynamics of individuals smaller than 50 mm diameter (but see [75] for a recent application of Leslie matrices on a full size range of *P*. *damicornis* in Taiwan). Through the use of demographic models, our study provides unique insight into the dynamics of juvenile coral assemblages and their influence to benthic community structure on Indo-Pacific coral reefs. While we did not explicitly explore site replication within habitats within our study, the frequent sampling periodicity (6 month time periods) and high within habitat quadrat replication (46 and 36 quadrats in the reef flat and slope, respectively) provided a unique insight into the fine scale dynamics of the smallest coral size classes that are often overlooked in previous studies of coral reef population dynamics. Our sensitivity analyses focussed on demographic parameters including recruitment, mortality, and growth of juvenile corals with three distinct life-history strategies in two common reef habitats. In general, the populations of weedy brooder, slow-growing massive, and fast-growing competitive corals showed similar sensitivities to perturbations within each habitat. Assemblages in the reef flat were most sensitive to perturbations on growth and mortality of larger size classes (i.e. 20–50 mm), whereas perturbations on the recruitment, growth and mortality of the smallest size classes (i.e. < 20 mm) were most sensitive on the reef slope. These results suggest that the different stressors that influenced the juvenile assemblages are habitat specific.

Three genera of brooders (*Isopora*, *Pocillopora*, *Stylophora*) with fast growth rates, and the slow growing massive *Porites* and Faviidae, were common to both habitats, yet these taxa did not increase coral cover. Corals on the reef flat are restricted in growth by physical constraints due to the height of water at low tides [7], which also drive extreme water temperature and chemistry [76]. There was a lack of available hard settlement space on the reef flat, and corals have to compete with fleshy macroalgae that dominate this habitat [39]. Therefore, the assemblage found on the reef flat may represent some kind of equilibrium for this environment that is dominated by weedy and stress tolerant taxa [38], communities that are common in degraded systems [28, 29, 77, 78]. In contrast, coral growth on the reef slope was not restricted by density-dependent space limitation or water height, and there was limited competition with fleshy macroalgae. The rapid growth of tabulate and digitate *Acropora* facilitated the increase in coral cover in the reef slope, and these taxa rapidly increase habitat complexity that supports many reef flora and fauna [4, 11, 79, 80]. The change in coral cover on the reef slope is still very much in a rapidly increasing trajectory due to the continual growth of tabulate and digitate *Acropora* colonies (C Doropoulos, *personal observation*), and large fluctuations in coral cover are common in this exposed reef slope habitat [7].

A positive stock-recruitment relationship (as observed in brooding corals) is a likely contributing factor to rapid recovery rates [81]. However, despite the increased density of recruit and adult brooders (*Isopora*, *Pocillopora*, *Seriatopora*, *Stylophora*) in both habitats, they hardly altered coral cover in this study. These taxa are highly opportunistic and have rapid generation times, but remain relatively small in size despite their rapid growth [28, 29]. It was broadcast spawning corals that drove the increase in coral cover on the reef slope, a pattern seen in corals that generated sigmoidal recovery in long-term studies of reef slope habitats in other Indo-Pacific ecosystems [8, 10, 11]. Acroporids are strong competitors that recruit to newly available space at high densities after annual mass spawning events [20, 21, 63, 82]. They use resources efficiently and invest their energy into rapid early growth to out-compete other individuals and dominate space [28, 79]. While our study did not find any stock-recruitment relationship with spawning corals, at broad spatial scales stock-recruitment in spawners can be a major contributor to reef recovery in isolated systems as has recently been shown in a remote atoll in north-western Australia [8].

Our study demonstrates how the complexities of recruiting coral assemblages can affect benthic habitat structure and trajectories. Specifically, in the system studied, stock-recruitment partially explained the recovery of brooder coral populations, but these taxa did not affect coral cover. Forces that constrain larval supply, the availability of optimal microhabitats, selective larval settlement, and differences in post-settlement survival all appear to have contributed to divergent recovery trajectories. In particular, recruit identity and life-history appear fundamental in driving the increase in coral cover found in Indo-Pacific reefs. The maintenance and recovery of coral populations may be seriously impaired if the recruitment of new individuals is strongly dependent on the size of the adult stock, and the positive stock-recruitment relationship suggests that the resilience of brooding corals is particularly threatened by both local and regional-scale disturbance. For broadcast spawning corals, however, it is more likely that only regional-scale disturbances would threaten recruitment [8, 83]. Unfortunately, those corals that exhibit the fastest recovery also happen to be some of the most susceptible species to large-scale perturbations including coral bleaching and disease [10, 29, 34, 84]. Therefore, understanding adult stock and the life-history of recruiting assemblages is fundamental to predict trajectories of benthic communities following small and large scale disturbances.

## Supporting Information

S1 Fig Multidimensional scaling (MDS) plot comparing the benthic community cover of the broad community in the reef flat (n = 10) and reef slope (n = 10), and the permanent plots in the reef flat (n = 46) and reef slope (n = 36), at the beginning of the study period (August 2009). Vector overlay represents correlations >0.6 based on Spearman ranking. Upward facing solid triangles = reef flat permanent plot; upward facing hollow triangles = reef flat broad community; downward facing solid triangles = reef slope permanent plots; downward facing hollow triangles = reef slope broad community. EAM = epilithic algal matrix; CCA = crustose coralline algae; MA = fleshy macroalgae.
(EPS)

S2 Fig Benthic community cover (mean % + SD) at the (a) reef flat and (b) reef slope habitats every six months, beginning in August 2009 and ending in February 2012. EAM = epilithic algal matrix; CCA = crustose coralline algae; Turf = dense turf algae; MA = fleshy macroalgae; Other = soft coral, sponge, *Tridacna*, unknown. Winter = August; summer = February (except summer 2011 = April).
(EPS)

S1 Table The number of settlement tile pairs retrieved and with recruits, and the number of new recruits at each sampling time from the reef flat and reef slope habitats. The summer sampling time was in February (or April in 2011) each year, approximately 3 months following the major annual spawning event. Each tile pair is 100 cm^2^.
(PDF)

S2 Table ANOSIM of the (a) global and (b) pair wise tests comparing the benthic community cover of the broad community in the reef flat and reef slope (n = 10), and the permanent plots in the reef flat (n = 46) and reef slope (n = 36), at the beginning of the study period (August 2009). (PDF)

S3 Table PERMANOVA results comparing the benthic community cover of the permanent plots between habitats (fixed) over time (random). There were 46 permanent plots on the reef flat and 36 on the reef slope. Monitoring began in August 2009 and continued every 6 months until 2012.
(PDF)

S4 Table ANOVA results comparing coral growth rates (mm per 6 months) among habitats (fixed) and coral taxa (fixed) over time (random). Monitoring began in August 2009 and continued every 6 months until 2012. Results are based on 999 permuations analysing the raw growth data that did not conform to homogeneity, therefore the α was set at 0.01 to avoid a type I error (Underwood 1997). Only significant post-hoc comparisons are displayed for the Ha x Ta interaction Massive = Mas; *Isopora* = *Iso*; Pocilloporidae = Poc; *Acropora* = *Acr*.
(PDF)

S5 Table ANOVA results comparing the coral growth rates (mm per 6 months) among and size classes (fixed) and taxa (fixed) over time (random). The recruits were separated into six size classes (I = 1–10, II = 11–20, III = 21–30, IV = 31–40, V = 41–50, and VI = >50 mm). Monitoring began in August 2009 and continued every 6 months until 2012. Results are based on 999 permuations analysing the raw growth data that did not conform to homogeneity for any effect, therefore the α was set at 0.01 to avoid a type I error (Underwood 1997). Only significant post-hoc comparisons are displayed for the Si x Ta interaction. Massive = Mas; *Isopora* = *Iso*; Pocilloporidae = Poc; *Acropora* = *Acr*.
(PDF)

S6 Table File of spreadsheets for all data collected during the study. (XLSX)
