# Supplementary material for: Linking Demographic Processes of Juvenile Corals to Benthic Recovery Trajectories in Two Common Reef Habitats
Source: PLoS One. 2015 May 26;10(5):e0128535. doi: 10.1371/journal.pone.0128535 (PMC4444195; doi:10.1371/journal.pone.0128535)
Supplement: S1 Table — The summer sampling time was in February (or April in 2011) each year, approximately 3 months following the major annual spawning event. Each tile pair is 100 cm2. (PDF) [file pone.0128535.s003.pdf]

Table S1. The number of settlement tile pairs retrieved and with recruits, and the number of new recruits at each sampling time from the reef flat and reef slope habitats. The summer sampling time was in February (or April in 2011) each year, approximately 3 months following the major annual spawning event. Each tile pair is 100 cm^2.

	Reef flat			Reef slope		
	Tiles retrieved	Tiles with recruits	New recruits	Tiles retrieved	Tiles with recruits	New recruits
Summer 2010	27	3	6	15	9	30
Winter 2010	3	2	4	8	2	3
Summer 2011	46	16	20	34	24	47
Winter 2011	17	4	5	22	3	5
Summer 2012	37	5	6	32	30	77
