# Supplementary material for: Linking Demographic Processes of Juvenile Corals to Benthic Recovery Trajectories in Two Common Reef Habitats
Source: PLoS One. 2015 May 26;10(5):e0128535. doi: 10.1371/journal.pone.0128535 (PMC4444195; doi:10.1371/journal.pone.0128535)
Supplement: S2 Table — (PDF) [file pone.0128535.s004.pdf]

Table S2. ANOSIM of the (a) global and (b) pair wise tests comparing the benthic community cover of the broad community in the reef flat and reef slope (n = 10), and the permanent plots in the reef flat (n = 46) and reef slope (n = 36), at the beginning of the study period (August 2009).

	R statistic	Significance %	Perms	Perms ≥ global R
a. Global test	0.538	0.1	999	0
b. Pair wise tests				
RF plot, RS plot	0.638	0.1	999	0
RF plot, RF broad	0.131	5.8	999	57
RF plot, RS broad	0.430	0.1	999	0
RS plot, RF broad	0.992	0.1	999	0
RS plot, RS broad	0.087	13.2	999	131
RF broad, RS broad	1.000	0.1	999	0
