# Supplementary material for: Linking Demographic Processes of Juvenile Corals to Benthic Recovery Trajectories in Two Common Reef Habitats
Source: PLoS One. 2015 May 26;10(5):e0128535. doi: 10.1371/journal.pone.0128535 (PMC4444195; doi:10.1371/journal.pone.0128535)
Supplement: S3 Table — There were 46 permanent plots on the reef flat and 36 on the reef slope. Monitoring began in August 2009 and continued every 6 months until 2012. (PDF) [file pone.0128535.s005.pdf]

Table S3. PERMANOVA results comparing the benthic community cover of the permanent plots between habitats (fixed) over time (random). There were 46 permanent plots on the reef flat and 36 on the reef slope. Monitoring began in August 2009 and continued every 6 months until 2012.

Source of variation	df	MS	Psuedo-F	*P* (perm)	Conclusions: pair-wise
Habitat	1	333780	332.37	0.001	
Time	1	18760	18.68	0.001	
Habitat x Time	5	6770	6.74	0.001	Habitat: Flat ≠ Slope at every time
Residual	488	1004			
