# Supplementary material for: Linking Demographic Processes of Juvenile Corals to Benthic Recovery Trajectories in Two Common Reef Habitats
Source: PLoS One. 2015 May 26;10(5):e0128535. doi: 10.1371/journal.pone.0128535 (PMC4444195; doi:10.1371/journal.pone.0128535)
Supplement: S4 Table — Monitoring began in August 2009 and continued every 6 months until 2012. Results are based on 999 permuations analysing the raw growth data that did not conform to homogeneity, therefore the α was set at 0.01 to avoid a type I error (Underwood 1997). Only significant post-hoc comparisons are displayed for the Ha x Ta interaction Massive = Mas; Isopora = Iso; Pocilloporidae = Poc; Acropora = Acr. (PDF) [file pone.0128535.s006.pdf]

Table S4. ANOVA results comparing coral growth rates (mm per 6 months) among habitats (fixed) and coral taxa (fixed) over time (random). Monitoring began in August 2009 and continued every 6 months until 2012. Results are based on 999 permuations analysing the raw growth data that did not conform to homogeneity, therefore the α was set at 0.01 to avoid a type I error (Underwood 1997). Only significant post-hoc comparisons are displayed for the Ha x Ta interaction Massive = Mas; *Isopora* = *Iso*; Pocilloporidae = Poc; *Acropora* = *Acr.*

Source of variation	df	MS	Psuedo-F	*P* (perm)	Conclusions: pair-wise
Habitat (Ha)	1	167.8	0.38	0.582	
Taxa (Ta)	3	4597.9	40.49	0.001	
Time (Ti)	4	1083.4	9.54	0.001	
Ha x Ta*	2	551.8	4.86	0.010	Habitat: all NS<br>Taxa: Reef flat – Mas < *Iso* < Poc<br>Taxa: Reef slope – Mas < *Iso,* Poc, *Acr* ; Poc < *Acr*
Ha x Ti	4	675.9	5.95	0.001	
Pooled[¶]	1552	113.5			

*Term has one or more empty cells

[¶]Pooled terms: Residual + Ta x Ti + Ha x Ta x Ti
