# Supplementary material for: Linking Demographic Processes of Juvenile Corals to Benthic Recovery Trajectories in Two Common Reef Habitats
Source: PLoS One. 2015 May 26;10(5):e0128535. doi: 10.1371/journal.pone.0128535 (PMC4444195; doi:10.1371/journal.pone.0128535)
Supplement: S5 Table — The recruits were separated into six size classes (I = 1–10, II = 11–20, III = 21–30, IV = 31–40, V = 41–50, and VI = >50 mm). Monitoring began in August 2009 and continued every 6 months until 2012. Results are based on 999 permuations analysing the raw growth data that did not conform to homogeneity for any effect, therefore the α was set at 0.01 to avoid a type I error (Underwood 1997). Only significant post-hoc comparisons are displayed for the Si x Ta interaction. Massive = Mas; Isopora = Iso; Pocilloporidae = Poc; Acropora = Acr. (PDF) [file pone.0128535.s007.pdf]

Table S5. ANOVA results comparing the coral growth rates (mm per 6 months) among and size classes (fixed) and taxa (fixed) over time (random). The recruits were separated into six size classes (I = 1-10, II = 11-20, III = 21-30, IV = 31-40, V = 41-50, and VI = >50 mm). Monitoring began in August 2009 and continued every 6 months until 2012. Results are based on 999 permuations analysing the raw growth data that did not conform to homogeneity for any effect, therefore the α was set at 0.01 to avoid a type I error (Underwood 1997). Only significant post-hoc comparisons are displayed for the Si x Ta interaction. Massive = Mas; *Isopora* = *Iso*; Pocilloporidae = Poc; *Acropora* = *Acr.*

Source of variation	df	MS	Psuedo-F	*P* (perm)	Conclusions: pair-wise
Size (Si)	5	443.7	4.14	0.001	
Taxa (Ta)	3	3881.1	36.22	0.001	
Time (Ti)	4	1855.4	17.32	0.001	
Si x Ta	15	459.3	4.29	0.001	Size: Mas – II < VI; *Iso* – all NS; Poc – I, II < IV, VI; III < VI; IV < VI; *Acr* – I < IV-VI; II < V, VI; III < VI; IV < VI; V < VI<br>Taxa: I – all NS; II – Mas < *Acr*; III – Mas < Poc, *Acr*; IV-V – Mas < *Iso,* Poc, *Acr*; VI - Mas < *Iso* < Poc < *Acr*
Pooled[¶]	1539	107.1			

*Term has one or more empty cells

[¶]Pooled terms: Residual + Si x Ti + Ta x Ti + Si x Ta x Ti
